# Students´ perception of interprofessional education in the bachelor programme “Interprofessional Health Care” in Heidelberg, Germany: an exploratory case study

**DOI:** 10.1186/s12909-018-1124-3

**Published:** 2018-01-25

**Authors:** Cornelia Mahler, Veronika Schwarzbeck, Johanna Mink, Katja Goetz

**Affiliations:** 1 Department of General Practice and Health Services Research, University Hospital Heidelberg, Marsilius-Arkaden, Im Neuenheimer Feld 130.3, D-69120 Heidelberg, Germany; 2 Institue of Family Medicine, Ratzeburger Allee 160, Haus 50, 23538 Lübeck, Germany

**Keywords:** Interprofessional education; student perception, Interprofessional competencies, Qualitative study, Interprofessional health care; focus group interviews

## Abstract

**Background:**

Interprofessional education is receiving increased attention worldwide. This has led to the development of a bachelor programme “Interprofessional Health Care” at the University of Heidelberg, Germany beginning in the winter semester 2011. Aim of this study was to evaluate the students’ perception of this innovative programme regarding interprofessional learning.

**Methods:**

An exploratory case study was conducted. A semi-structured guideline was developed and seven focus groups were performed with the students of the first three cohorts in 2012–2014. Data was transcribed and analyzed using content analysis leading to main categories, one of which was titled “interprofessional learning”. This article presents the results focussing on the students’ experiences regarding interprofessional education and learning during their first two semesters of the programme.

**Results:**

Four main categories related to interprofessional learning were developed inductively. Students assessed “interprofessional learning” in general as positive and wished to encounter a more intense experience and collaboration with different health professions during their studies. Students reported to benefit from the programme due to a better understanding of other professions and their different perspectives. They described decreased hesitance to approach other health professions in every day practice. Results are in line with the four domains of the Interprofessional Core Competencies.

**Conclusion:**

All in all students at an early stage recognized the benefit of interprofessional learning for their studies and their everyday work in practice showing the way forward for the bachelor programme and encouraging more interprofessional encounters with students from other health care programmes.

**Electronic supplementary material:**

The online version of this article (10.1186/s12909-018-1124-3) contains supplementary material, which is available to authorized users.

## Background

Interprofessional education and interprofessional learning have received increased attention worldwide in the last decade as a number of patient and health services related problems may be improved by better collaboration between health professionals. The expert advisory board of health care in Germany recommends improving collaborative working in the interprofessional context in order to provide more effective healthcare [1]. Thus, interprofessional education is needed and “occurs when students from two or more professions learn about, from and with each other to enable effective collaboration and improve health outcomes.” ([2], p. 10). In 2008 the WHO study group on Interprofessional Education on Collaborative Practice identified that interprofessional education may be existent in many of the 42 investigated countries, but in most of them there is no standard guideline for interprofessional teaching. Still, it was perceived as quite useful, as “respondents reported that they had experienced many educational and health policy benefits from implementing interprofessional education.” ([2], p. 17). In countries like Canada, UK, Sweden or Denmark joint faculties for Health Sciences have been offering interprofessional education for nearly twenty years, whereas in others it is only just evolving [3, 4].

Students’ perception and experience of interprofessional education play a relevant role in the introduction on interprofessional education and have been analysed in various programmes. A study of the students’ perspective on interprofessional learning conducted by Pollard et al. in Great Britain showed that the attitude of students towards interprofessional learning and working, though becoming more negative throughout their education, was mainly positive. In general the more mature and experienced students are more positive about interprofessional education than the younger ones. The authors also conclude that, according to their findings, interprofessional education reduces stereotypes of different professions [5]. Other studies in English speaking countries have shown that students who have gone through interprofessional training have recognized its importance for patient care [6] and have improved interprofessional skills [7, 8]. In addition interprofessional educational initiatives were valued by graduates [9], contributed to a better understanding of professional roles [10] and even a brief intervention had positive outcomes on attitudes towards interprofessional teams and learning [11] .

In Germany, traditionally, medical training is university based while other health professions (nurses, therapists etc.) are still predominately trained in vocational degrees and not in academic programmes [12]. An innovative approach to develop an interprofessional undergraduate bachelor degree was established as collaborative programme between the University of Heidelberg and the Heidelberg Academy for Health Professions [13] giving the opportunity to achieve a vocational and the bachelor degree “Interprofessional Health Care” in parallel. To study this programme students begin with their vocational training at the Heidelberg Academy for Health Professions in their chosen field (geriatric, general and paediatric nursing, physiotherapy, speech and language therapy, midwifery, orthoptics, medical technical laboratory assistance or medical technical radiography assistance) and enrol for the bachelor programme within their first year of training. The modules in the programme were developed by an interprofessional expert team and address the competencies needed for inteprofessional practice as recommended by the WHO (2005) as well as academic skills and competencies for evidence based practice [13]. Whilst students remain in their “monoprofessional” cohorts during vocational training, the modules delivered by the university in the bachelor programme address an interprofessional cohort which is comprised of students of potentially all health care professions mentioned above. The students study together over a period of four years using methods of “learning in common” and “common learning” [14]. The programme has established a close collaboration with the medical school of the Medical Faculty of the University of Heidelberg and selected seminars are conducted collaboratively if appropriate and where logistical barriers have been managed [15]. The first collaborative seminar takes place within the first two semesters of the programme [16].

As no expert knowledge/research on interprofessional education nor on students’ perception of interprofessional learning in Germany were available when the programme started, it was deemed necessary to gain insight on how this novel concept was received. The aim of this explorative case study was in particular to report on and to gain insight in the students’ perspective on interprofessional learning in general within this new programme.

## Methods

### Study design

A qualitative approach in form of an exploratory case study [17] was regarded as suitable to describe and analyse the students’ perception of this novice field in Germany. This approach was regarded as suitable as an empirical case study investigates “contemporary phenomenon in its real-life context, especially when the boundaries between phenomenon and context are not clearly evident” ([17], p.16). The first two semesters of the programme Interprofessional Health Care were considered as the ‘defined unit’, which were researched within the ‘defined context’ of the whole bachelor programme. Focus groups were performed in order to gain in-depth insight on students’ perspective of interprofessional learning.

### Study setting

All the focus group interviews took place in the Department of General Practice and Health Services Research in Heidelberg, Germany, the administrative building of the bachelor degree programme. The interviews were performed at the end of the summer semester in 2012, 2013 and 2014, by three interviewers (KG, VS & JM) who had not been involved in teaching in order to allow students to speak freely.

### Subjects

All students enrolled in the bachelor of science programme “Interprofessional Health Care” at Heidelberg University were invited to participate. At the time of the interview they had all finished the second semester of their academic programme and in parallel were still in their vocational training programme. Students interviewed in July 2012 (Cohort 2011, *N* = 23) were members of the first cohort of the university programme that started in winter semester 2011. One year later the cohort of 2012 (*N* = 19) was interviewed in July 2013; the cohort of 2013 (*N* = 18) in July 2014. The interviews were conducted as part of the overall programme evaluation. Participation in the focus group interview was voluntary. Written consent was obtained from each student before the focus groups were conducted. No personal information was collected.

### Data collection

A semi-structured guideline was developed and slightly adapted in wording for easier comprehension of the focus group interviews for each cohort. The guideline addressed the first year of the bachelor programme. To show details the guideline has been added as an additional file [see Additional file 1]. When discussions emerged between the participants during the focus groups, the interviewer served as a moderator and facilitated the process. Sometimes the interviewer had to address the students individually one after the other in order to receive information. Overall seven focus groups with 49 students were held, with five to nine students in each group, following the semi-structured guideline. The focus groups lasted between 50 to 90 min.

The focus groups were audio- and video recorded and transcribed verbatim. Video recordings were used to support transcription. Transcripts were de-identified and videos were deleted after transcription. Primary purpose for these focus groups was for quality assurance to receive immediate feedback on outcomes and acceptance of the new innovative interprofessional programme and to adapt where necessary. Ethical approval was not required for this study according to the Ethics Committee in Heidelberg.

### Data analysis

Two researchers (VS & JM) analyzed the transcripts after each run independently by first paraphrasing all statements and then clustering them to meaningful categories. Categories and subcategories were generated inductively by each researcher. Data was compared regularly throughout the analysis process and consensus was reached. Exemplary quotations from the original data were picked to underpin each category. In a next step the research team (CM, VS & JM) compared all categories of the three cohorts, looking for similarities, differences and developments between the cohorts within the data. New universal categories were identified that matched the hitherto gathered information. For publication the student quotations were translated from German into English.

## Results

### Description of the study sample

The study sample consisted of 49 students who participated in the focus groups. The 17 students of the first cohort (2011) were divided into three focus groups (two with 5 students (FG 2.2011 & FG 3.2011) and one with 7 students (FG 1.2011)). In 2013 two focus groups (FG 1.2012 & FG 2.2012) with 7 students each were performed. Two focus groups (FG 1.2013 with 9 and FG 2.2013 with 7 students) took place in 2014. Participants of every group were roughly the same age, stage of vocational training and university education however their health profession differed (see Table 1). The following professions were represented: pediatric nursing, general nursing, geriatric nursing, medical technical laboratory assistance (MTLA), medical technical radiography assistance (MTRA), speech therapy, orthoptics and physiotherapy.

**Table 1:** Gender and health professions interviewed in each cohort

	Gender (m/f)	Professions interviewed
Cohort of 2011 (3 Groups)	16 female 1 male	General nursing, pediatric nursing, geriatric nursing, orthoptics, medical technical laboratory assistants and medical technical radiography assistants,
Cohort of 2012 (2 Groups)	9 female 4 male	General nursing, pediatric nursing, medical technical laboratory assistants, physiotherapy, speech therapy, orthoptics
Cohort of 2013 (2 Groups)	14 female 2 male	medical technical laboratory assistants, general nursing, physiotherapy, speechtherapy

### Presentation of categories

Six main-categories were identified in the seven focus groups, namely “general feedback about the first year”, “compatibility”, “interface to vocational training”, “role identification”, “future perspectives” and “interprofessional learning”. This article will demonstrate the results of the main-category “interprofessional learning” which included four sub-categories (see Table 2).

**Table 2:** 1st and 2nd level categories of the main category “interprofessional learning”

Main category: “Interprofessional Learning”	
1st level-sub categories	2nd level sub categories
Benefits	Learning about each other Overcoming prejudice Learning from each other Practical collaboration
Challenges	Different levels of medical knowledge Mixing of professions Equality between professions
Atmosphere within the group	Contact between students Mutual support within the group Group work Development of group atmosphere
Interprofessional learning with medical students	Organizational aspects Future perspectives on further seminars Participation of medical students

In the category “interprofessional learning” all student statements concerning collaborative learning with students from other health care professions were taken into account. “Benefits”, “challenges”, the “atmosphere within the group” as well as “interprofessional learning with medical students” were identified as sub-categories.

Most of the students saw more benefits than challenges regarding interprofessional learning. The most important advantage that students of all three cohorts described was a more distinct knowledge and understanding of other students’ professional practice, as well as the knowledge of formerly unknown health professions, which they gained in seminars as well as informally in chats on the side with fellow students. “Now I have a better understanding of the other health professions, even in practice, in my daily work. I understand, when someone… when something goes wrong, because I know the whys and wherefores. And I think, this is in favour for me now.” (FG 2.2011, PP04).

The understanding of other health professions helped them understand different practical working approaches. Some students pointed out, that they perceived an evolved understanding for each other as a result of interprofessional learning. As a consequence students of all three cohorts stated that they took benefit from the chance to become aware of and overcome prejudices “And this is how you get to know the other professions and you lose this (…) this wearing of blinders, this pigeonholing.” (FG 2.2012, PP 06).

In addition learning from each other was another advantage described in all three cohorts. Students reported that they took benefit from the knowledge of other health professionals as it complemented their own expertise. They saw enrichment and broadening of their own perspectives resulting from an exchange on others' point of view:“I think, this is what this programme mounts up to, namely, this interprofessional thing. Up until now we learn what the others, what they do in their health professions and we get personal impressions, not quite, one does not learn the profession but we can exchange views with the other students.” (FG 1.2013; PP03).

Concerning the practical relevance of the interprofessional education all students recognized the importance of interprofessional cooperation, communication and interface management. They described a benefit for their practical work due to the perceived reduction of reservations towards different professions. Students of the second and third cohort described how an increased open-mindedness lead to a better interprofessional collaboration in practice and thus to a more holistic patient care. “Speaking for myself, I kind of realized how important the support of other professions is, for example, being on the ward or something, (…) I am strongly dependent on the support of the nurse and the other way around.” (FG 2.2012; PP 05).

Some students described different levels of medical knowledge as a difficulty in interprofessional learning. “But I think sometimes it is difficult with the content (…). Sometimes, however it happens, that you notice, okay, one part of the group is absolutely bored, because they have already heard it a thousand times. The others are following with great interest, or, respectively, still have their difficulties in understanding. Thus I think it is perceivable that there are differences.” (FG 1.2011, PP05).

They also saw a challenge in mingling with students of other health professions, as they tended to stay in their own peer health professional group: “… even during the seminars one stays within one’s own professional group, that is, also concerning our order of seating. But in my opinion at the beginning, especially in the first semester, … build, for example, expert groups or something like that and then create more exercises with reference to interprofessional collaboration.” (FG 2.2011, PP03). It became clear that the interprofessional group constellation did not emerge automatically but some students expressed the wish to be mixed up by the teaching staff in order to engage more with the other professions.

Mainly students of the first cohort complained about examples and content of the class addressing predominately one health profession. They noticed that it was a challenge for the teaching staff to address all professions at the same time or at least equally, with the nursing profession being the largest group. “And I think as a teacher it is not possible to cope with all professions equally.” (FG 1.2011 PP 04) This was mentioned less often in the second cohort, where only one student expressed the wish for more heterogeneity in the group because of nursing dominance. The third cohort did not address this issue any more.

There were some single statements addressing different challenges. One student seemed to find it hard to develop a common language, aside from all professional technical terms and one pointed out that they might be learning together, but they lacked learning from each other. In the first cohort two medical technical laboratory assistant students described difficulties based on missing relevance of interprofessional learning for their practical work, as they had little contact to other health professions in their professional practice.

Regarding the group atmosphere, students described a loose contact within the group, but they also pointed out the need for spending more time with their fellow students in order to get to know each other better. This request was expressed less intensely by students of the first cohort. “I think it is easier, at least in my case, to be in a group with all the nurses. Sometimes it depends on the topic, but in case we get a medical text or something (…) for me being in a group with nurses then it is easier compared to working together with an orthoptist.” (FG 1.2011, PP01).

The first two cohorts described a positive atmosphere in the group due to the possibility to exchange views about their shared concerns regarding the new study programme. Consequently, they profited from mutual support and some of them declared a strong alliance. Students of the second cohort reported an improved atmosphere in the group through obligatory group work with students of other professions, opposing their inclination of sticking together with students of the same profession. “I think it came from (…) Ms. B. did mix up all numbers and name tags and distributed them randomly, so that we came to sit together with people we did not use to have a lot of contact with, and I think that broke the ice in the end.” (FG 1.2012, PP 01) Another reason for the improved atmosphere was seen in the increasing amount of time they spend together. Furthermore, single statements reasoned this improvement with the evolvement of study groups in order to learn together, as well as with increased shared activities in their spare time. Interesting discussions in breaks were mentioned as more effective to get to know each other than the shared lessons.

Most students reported that they enjoyed the interprofessional courses with the medical students. Nevertheless most of the students complained that neither they nor the medical students were informed about this interprofessional setting beforehand. They therefore expressed their wish to standardize these courses, including information for all participants. Furthermore students expressed interest in more shared lectures with medical students and would appreciate a higher participation of medical students. “I thought it was really good. Maybe there were only a few, I think that in the second semester only two [medical students] were left. But they were really nice and interested.” (FG 1.2013, PP 05).

## Discussion

The aim of the explorative case study was to evaluate students’ perspective on the bachelor degree “Interprofessional Health Care”, focussing on their perception of the interprofessional learning and setting which the programme offers. Results demonstrated that interprofessional learning is perceived positively by the students at this early stage in their studies and was associated with benefits and challenges. A positive interprofessional atmosphere within the group was perceived and the wish to engage more with medical students was stated.

Aspects regarding heterogeneous groups and different levels of knowledge have been described in the interprofessional health care literature [14, 18]. To overcome these challenges didactical approaches have been established in the programme such as implementation of group approaches in which more knowledgeable students are able to assist their peers [16].

Although initially not targeted during planning of the first focus groups, our results are very much in line with the framework of the *Core Competencies for Interprofessional Collaborative Practice*, a report sponsored by the interprofessional education collaborative [19] (Interprofessional Education Collaborative [IPEC], 2011). Our results show that all four competency domains are adressed during the first two semesters and students gain IP competencies within the first year of the programme.

Within the first competency domain 1 “Values/Ethics for interprofessional practice” (VE), which describes the “Work with individuals of other professions to maintain a climate of mutual respect and shared values” ([19], p.19), students show an evolving understanding for each other and for overcoming prejudices. They talk about a better understanding of their fellow students’ practice field, which they gain insight in not only in shared lectures but also in private conversations. This shows that they are able to “respect the unique cultures, values, roles/responsibilities, and expertise of other health professions.”(VE4) ([19], p. 19) Becoming aware of one’s preconceptions can be seen as a precondition to “embrace the cultural diversity and individual differences that characterize (…) the health care team”.(VE3) ([19], p. 19) The fact that the students express a benefit from communicating about troubles with their fellow students suggests that they feel respected by the others.

A number of the descriptors among the second competency domain: Roles / Responsibilities: “Use the knowledge of one’s own role and those of other professions to appropriately assess and address the healthcare needs of the patients and populations served” ([19], p. 21) are addressed by the students. They get to know the roles and responsibilities of the other health care professions, what enables them to decide which health care profession they can consult in practice. A competency which is not addressed at this stage of the programme is the recognition and definition of one’s own roles, responsibilities, limitations and abilities. This is a competency domain which needs more attention within the early stage of the programme. However, former studies come to the conclusion that students at an early stage in their training are often not fully aware of this competency [5, 20, 21]. Maybe the missing statements on personal skills and knowledge are a consequence of the type of data collection in form of a group interview: In a focus group it is not the individual who is in the primary focus but the whole group and their interactions. Thus the students might not have seen a necessity in expressing their opinion about their own skills.

Interprofessional communication (Competency domain 3: “Communicate with patients, families, communities, and other health professionals in a responsive and responsible manner that supports a team approach to the maintenance of health and the treatment of disease.” ([19], p. 23)) still seems to be a bit difficult for the students, as at first, they tend to stay in groups of their own profession. Secondly, one student even complains about the lack of a common language that can be understood independent of expertise and knowledge. Still, as said before, they seem to be able to communicate about problems and prejudices, about the different work fields and about different viewpoints. Some students note that the conversations in the breaks are more effective, which might show that communication on a personal level is no problem, whereas communication on interprofessional level is still perceived as difficult at this stage. But this could also be due to the fact that the students do not have that much practical experience. Intergroup contact theory [22] underpins these findings pointing out that mere emersion with other groups facilitate change and are more likely to lead to more positive attitudes toward one another under favourable conditions such as equal status and situations in which social norms support equality [23, 24]. Communication with patients was not mentioned at all.

Regarding Team and Teamwork (Competency domain 4: “Apply relationship-building values and the principles of team dynamics to perform effectively in different team roles to plan and deliver patient-/population- centred care that is safe, timely, efficient, effective, and equitable.” ([19], p. 25)) some students mentioned the improvement of the atmosphere within the group as a result of more interprofessional group work, based on the statements about the positive experience in learning and working together. However, some of the competencies of this domain were not mentioned at all. This might be due to the fact that team functioning was not quite the topic of the focus groups. Furthermore the contents the students learn in their first three semesters are rather theoretical and, as some of them have said, not all of them can see a practical relevance. At the time the focus groups were held the students only saw each other once a week and did not really work together but only learned together in an interprofessional team.

The overall results of our study confirm findings of the 2010 WHO study group on interprofessional education in 42 different countries [2] as well as the results of Pollard et al., as the Heidelberg students, also describe the learning about other profession as beneficial in reducing prejudices [5] .

Benefiting from each other’s perspective and getting to know the different professional practice, are mentioned as advantages that occurred. Apparently students slowly experience a change from staying in their own professional group, due to feeling safer, to showing interest in other perspectives and being curious about each other’s profession. The benefits regarding a better practical collaboration seem to be due to a better understanding and awareness of challenges that the other health professions face [25]. Through this awareness students can be more empathic with other professions and seem to lose the fear of interacting and working together as a team. Analysing the students’ statements it became clear that learning from and about each other were predominantly seen as benefits of the interprofessional education whereas learning with each other still provided some challenges concerning the heterogeneity of the group. This is possibly due to social identity of the students at this early stage of their vocational training where their main social group of interest is the professional group [26].

In this case students see challenges in mixing up with fellow students from different health professions. It seems that students in the early part of the programme tend to stay in their own professional (peer) group, which might be a result due to the different professionally spoken languages, perceived stereotypes and socialization, this also confirming aspects of intergroup contact theory [22]. Pollard et al. have come to the same conclusion, pointing out: “that professional socialization is a particularly strong influence on students’ attitudes to collaborative learning and working.” [5](2005, p. 265) Furthermore, it could be that the students have problems to overcome obstacles to communicate with each other, since there could be a feeling of being different in the beginning, which seems to decrease over time. In addition, students seem to need help overcoming the fear of contact, for example by teachers deciding about group constellation.

Although they describe difficulties due to the different levels of knowledge, the advantages seem to outweigh. Challenges relating to the inequality of referring to all professions the same way could lead to a different degree of identification with the group, as well as with the study programme itself.

In reference to the atmosphere in the group, the cohort of 2011 showed less interest in getting to know their classmates better outside the classroom. This could be due to their pioneer status as the possibility of enrolling in the programme was not known to them until shortly before the programme started.

Concerning the interprofessional lectures with the medical students, almost all students reported that they enjoyed these courses. A few students pointed out, that they also liked this kind of interprofessional lecture in order to prove to themselves to be as competent as medical students. This could be an important aspect regarding different hierarchical levels in the German health system. They might see an opportunity to overcome hierarchy through shared lectures with medical students.

### Strengths and limitations

A strength of the study is that the focus group interviews were conducted and predominately analysed by independent interviewers and researchers in order not to bias the results in any way.

A limitation can be seen in the relatively small number of students and that the interviews were only performed at one site and a designated programme leading to a selection bias and limitations regarding transferability of results to other programmes. However, the results give good insight in student’s perception of this programme and further direction for improvement of the interprofessional education within the programme.

## Conclusion

In conclusion, the study demonstrates the benefits of the interprofessional programme from a student perspective and encourages us to continue and further develop the bachelor programme “Interprofessional Health Care”. Students reported the need for improving interprofessional learning activities at this early stage of the programme. However, looking back on the three consecutive cohorts development in students’ attitude towards learning in an interprofessional group as well as their confidence in studying interprofessionally can be recognized. These students have already recognized the benefit of interprofessional learning and of their open-mindedness towards other professions in their everyday work. Recognizing prejudices and stereotypes that are prevalent in the health system, students will discover ways to overcome these. They will encounter their co-workers in an open manner and will be able to develop a better mutual understanding. We are confident that this degree will contribute to a workplace ready health professional workforce which will foster interprofessional collaboration.

## Additional file

Additional file 1: Interview Guideline. Semistructured interview guideline for the focus groups. (DOCX 17 kb)

## Abbreviations

FG: Focus group
MTLA: medical technical laboratory assistance
MTRA: medical technical radiography assistance
PP: Participant
VE: Values/Ethics for interprofessional practice,
