# Supplementary material for: Students´ perception of interprofessional education in the bachelor programme “Interprofessional Health Care” in Heidelberg, Germany: an exploratory case study
Source: BMC Med Educ. 2018 Jan 25;18:19. doi: 10.1186/s12909-018-1124-3 (PMC5785847; doi:10.1186/s12909-018-1124-3)
Supplement: Additional file 1: — Interview Guideline. Semistructured interview guideline for the focus groups. (DOCX 17 kb) [file 12909_2018_1124_MOESM1_ESM.docx]

**Semi structured guideline for focus groups**

| **Category** | **Question** | **Time (Minutes)** |
| --- | --- | --- |
| Introduction | General information | 5 |
| Question 1 | **Evaluation of the first module**  When you look back on the last 12 months   - *What content was very relevant / important to you?* - *Where do you see possible room for improvement?* | 10 |
| Transition | The programme combines vocational training and an academic qualification. How do you cope with this double burden. |  |
| Question 2 | **Compatibility / Overlaps**   - *Which difficulties and challenges do you encounter in the compatibility of academic and vocational training?* - *Which overlaps in content do you see? Helpful? Unnecessary?* | 15 |
| Transition | At the moment you are in the role of a trainee and a student. | 2 |
| Question 3 | **Individual role perception**   - *In which situations do you feel like a student?* - *Please report on the feedback you receive from your social environment (friends, family)* - *Please describe what experiences you make in the practice setting.* | 20 |
| Transition | In the seminars of the programme you learn together with students from other health professions. | 2 |
| Question 4 | **Concept of interprofessional Learning**   - *How do you perceive learning together with other health professionals? Benefits?, challenges?, Learning together with medical students?* - *Professional identity: in what way has the perception of you own (and other) health profession(s) changed.* | 15 |
| Wrap-up | - *What are your expectations and worries about the future?* - *What are you looking forward to and what are your concerns?* | 10 |
|  |  | 64 |
